# Local causal dynamic integrated global mode guidance transformer network for pedestrian trajectory prediction

**DOI:** 10.1371/journal.pone.0347049

**Published:** 2026-04-20

**Authors:** Sunwei Gong, Yinxin Bao, Yingyan Hou, Wanxuan Lu, Quan Shi

**Affiliations:** 1 School of Transportation and Civil Engineering, Nantong University, Nantong, Jiangsu, China; 2 Target Key Laboratory of Cognition and Application Technology and Key Laboratory of Network Information System Technology, Aerospace Information Research Institute, Chinese Academy of Sciences, Beijing, China; 3 School of Information Science and Technology, Nantong University, Nantong, Jiangsu, China; UAEU: United Arab Emirates University, UNITED ARAB EMIRATES

## Abstract

Pedestrian trajectory prediction is crucial for autonomous vehicles, which face challenges in integrating complex spatiotemporal dynamics, managing multi-modal future behaviors, and ensuring real-time performance. This paper introduces the Local-Global Collaborative Transformer Network (LGCMT) to address these issues. LGCMT features an innovative local-global collaborative encoder comprising two key modules: a Sparse Causal Temporal Attention (SCT-MSA) module, designed to extract fine-grained local causal dynamics, and a Global Context Encoder that utilizes Cosine Similarity Attention to capture macro-level spatiotemporal patterns. For multi-modal prediction, LGCMT employs a parallel Non-Autoregressive (NAR) decoder guided by a motion pattern library, which efficiently generates diverse trajectory candidates covering key future likelihoods. Extensive evaluations on the standard ETH/UCY benchmarks and the large-scale Stanford Drone Dataset (SDD) demonstrate LGCMT’s robust performance. On ETH/UCY, the model improves ADE and FDE by approximately 4.8% and 5.6% compared to the competitive TUTR baseline. Moreover, the proposed framework achieves exceptional inference efficiency, establishing LGCMT as a potent solution that effectively balances accuracy, multi-modality, and operational speed for real-time applications.

## 1 Introduction

Accurate and efficient pedestrian trajectory prediction is essential for safe autonomous navigation [1,2] and effective human-robot interaction [3]. In dynamic environments, decision-making systems must anticipate future states by leveraging historical motion patterns and social cues [4]. Although deep learning has substantially advanced predictive performance, real-world deployment still hinges on addressing a key trade-off: achieving strong representational capacity to capture complex spatiotemporal dynamics while maintaining inference efficiency to satisfy real-time latency requirements.

Modeling pedestrian motion faces two structural challenges regarding feature representation and output generation. First, pedestrian movement is governed by two distinct temporal scales: local causal dynamics, representing immediate kinematic reactions to surroundings, and global motion trends, representing consistent long-term directionality. Existing architectures often struggle to balance these. For instance, Transformer-based models like AgentFormer [5] utilize dense self-attention across the full sequence, which entangles local and global cues within a unified attention map rather than explicitly separating them. Conversely, methods like STAR [6] process spatial and temporal dimensions via separate stages. While this design is structured, it lacks an explicit mechanism to disentangle multi-scale temporal dynamics within its processing pipeline.

**Fig 1:**
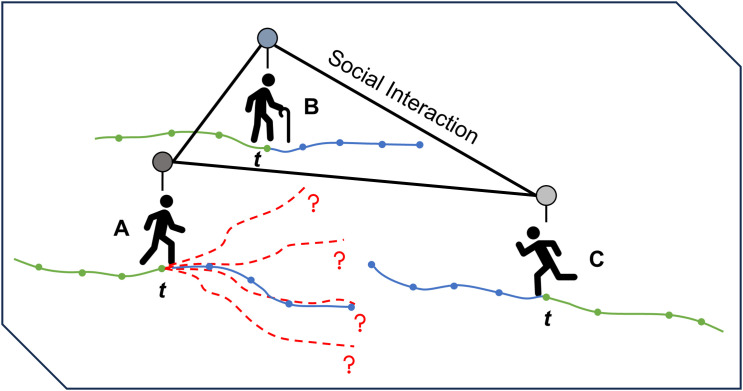
Social interactions induce multi-modal future uncertainty. Given the same observed history, different interaction outcomes can lead to multiple plausible future trajectories (red dashed), while only one is realized as the ground truth (blue). This illustration motivates the need to predict multiple modes to capture the eventual outcome.

Second, the inherent multimodality of human behavior—where a single history can lead to multiple plausible futures—demands modeling a distribution of possible trajectories. As illustrated in Fig 1, social interactions create diverse plausible paths from the same observed history. However, generating these hypotheses efficiently remains difficult. Generative approaches based on diffusion models [7] or GANs [8] often incur high computational overhead due to iterative denoising or complex sampling. Among Transformer-based methods, high-fidelity approaches frequently rely on autoregressive (AR) decoding [5,9], which requires sequential forward passes proportional to the prediction horizon *T*_pred_. This linear scaling of latency with forecast length poses a significant challenge for safety-critical, real-time applications.

To address these limitations, we propose the LGCMT, a framework designed to balance structural efficiency with predictive diversity. LGCMT introduces a dual-branch encoder to explicitly model the hierarchy of motion: a SCT-MSA branch captures short-term, history-dependent kinematic adjustments, while a Cosine Similarity Attention branch extracts macro-level directional trends. To ensure scalability in crowded scenes, we incorporate a distance-based spatial filtering strategy that reduces interaction complexity from quadratic to linear.

For decoding, we replace the standard autoregressive loop with a Library-Guided Non-Autoregressive (NAR) decoder. By retrieving structured motion patterns from a library learned on training trajectories, the model generates a diverse set of candidates in a single decoding pass, reducing the number of forward passes from *T*_pred_ to 1.

Our specific contributions are:

1. **Local-Global Collaborative Encoder:** We propose a specialized encoder that disentangles local causal dynamics from global motion trends using parallel sparse-causal and cosine-similarity attention mechanisms. This design explicitly separates immediate reactions from long-term directionality at the attention level.
2. **Library-Guided NAR Decoding:** We introduce a parallel prediction framework that utilizes a learned motion pattern library to guide non-autoregressive generation. This approach eliminates the latency of sequential decoding and avoids the iterative overhead associated with diffusion-style models.
3. **Efficiency and Accuracy Balance:** Validated on ETH/UCY and the large-scale SDD benchmarks, LGCMT achieves competitive accuracy compared to recent baselines. It demonstrates exceptional inference speed (approximately 2.8 ms per sample), making it highly suitable for real-time deployment in complex environments.

## 2 Related work

This section reviews existing literature focusing on two critical dimensions: spatiotemporal representation learning and the trade-off between multimodality and inference efficiency.

### 2.1 Spatiotemporal representation and interaction modeling

Early data-driven approaches utilized Recurrent Neural Networks (RNNs) to model sequential dependencies. Social-LSTM [10] introduced social pooling to aggregate neighbor information, a concept refined by subsequent attention-based RNNs [11,12]. While Transformers have gained traction, RNN architectures continue to evolve; for instance, the recent AFC-RNN [13] incorporates adaptive forgetting controllers to explicitly manage historical redundancy, demonstrating the continued relevance of recurrent structures for temporal modeling.

Graph Neural Networks (GNNs) [14] offer a flexible topology for modeling interactions, treating agents as nodes. Approaches like SGCN [15] and Social-STGCNN [16] leverage sparse graph convolutions to capture social effects. However, GNNs typically prioritize spatial topology, often employing simpler temporal aggregation mechanisms compared to attention-based sequence models.

The Transformer architecture [17] addresses long-range dependencies via self-attention. Early adaptations like STAR [6] interleaved spatial graphs with temporal Transformers. More recent works focus on enhancing robustness via specific constraints or additional modules. For example, TP-EGT [18] introduces a collision-aware Graph Transformer within a multi-task framework to explicitly predict interaction probabilities. Similarly, APT-TP [19] utilizes semantic maps and inverse reinforcement learning to enforce fine-grained trajectory-scene consistency. These constraint-aware Transformers primarily add supervision signals, modules, or extra inputs on top of Transformer backbones, instead of explicitly separating local causal dynamics and global trends at the attention level. In contrast, our LGCMT focuses on the intrinsic efficiency of the attention structure itself, employing sparse causal attention to strictly model the arrow of time for local dynamics, distinct from global trend analysis.

### 2.2 Multimodality and inference efficiency

Pedestrian trajectory prediction is inherently multimodal. Generative Models handle this by learning latent distributions. GANs [3,8] and CVAEs [20,21] sample from latent noise to generate diverse paths. Recently, Diffusion Models [7,22] have achieved high fidelity in distribution modeling but typically require multiple reverse steps for denoising, increasing inference cost. Normalizing Flows [23] offer exact likelihood estimation but often involve complex invertible transformations.

Deterministic Multi-Hypothesis approaches offer an alternative. Many Transformer-based models, such as AgentFormer [5], employ Autoregressive (AR) decoding to generate multimodal distributions. While AR ensures temporal coherence, it requires *T*_*pred*_ sequential forward passes, creating a latency bottleneck. Non-Autoregressive (NAR) approaches, such as query-based set prediction methods like TUTR [24], attempt to generate all time steps simultaneously. LGCMT extends this NAR paradigm by conditioning predictions on a discrete, learned motion pattern library. This structured guidance aims to ensure trajectory plausibility without the computational overhead of iterative sampling or sequential decoding loops.

## 3 Materials and methods

This section elaborates on the LGCMT, a model developed to address key challenges in pedestrian trajectory prediction. The proposed framework integrates specialized encoding, multi-mode hypothesis generation, and parallel decoding mechanisms. Subsequent subsections detail the problem formulation, the overall architecture, the design of each core component, and the training methodology.

### 3.1 Problem formulation and model overview

The objective of pedestrian trajectory prediction is defined as follows: Given the observed historical position sequence for a target pedestrian *i* over the past *T*_obs_ time steps, denoted as $X_{i}=\{p_{\left(i,t\right)}\in {\mathbb{R}}^{2}|t=1,\ldots ,T_{\text{obs}}\}$, and considering the historical trajectories $X_{j}=\{p_{\left(j,t\right)}\in {\mathbb{R}}^{2}|t=1,\ldots ,T_{\text{obs}}\}$ of neighboring pedestrians $j\in {𝒩}_{i}$. To efficiently handle crowded scenes and filter out irrelevant interactions, we explicitly define the neighbor set ${𝒩}_{i}$ based on a fixed spatial radius rather than considering all pedestrians in the scene. Specifically, a pedestrian *j* is included in ${𝒩}_{i}$ if and only if their distance to the target *i* is within a threshold τ:


$${𝒩}_{i}=\{j\in 𝒫⧵\{i\}|\|p_{\left(i,t\right)}-p_{\left(j,t\right)}{\|}_{2}<\tau \},$$ (1)

where 𝒫 denotes the set of all pedestrians in the scene. This distance-based filtering strategy effectively reduces the computational complexity from quadratic relative to the crowd size to linear relative to the number of relevant neighbors, preventing noise from distant agents and ensuring scalability in dense environments.

The task is to predict a set of *K* plausible future trajectories for pedestrian *i* over the next *T*_pred_ time steps. This output set is represented as ${𝒴}_{i}=\{Y_{\left(i,m\right)}|m=1,\ldots ,K\}$, where each individual trajectory hypothesis $Y_{\left(i,m\right)}=\{{\hat{p}}_{\left(i,t\right)}\in {\mathbb{R}}^{2}|t=T_{\text{obs}}+1,\ldots ,T_{\text{obs}}+T_{\text{pred}}\}$ signifies a distinct future path. This formulation inherently accommodates the multi-mode nature of pedestrian movement.

**Fig 2:**
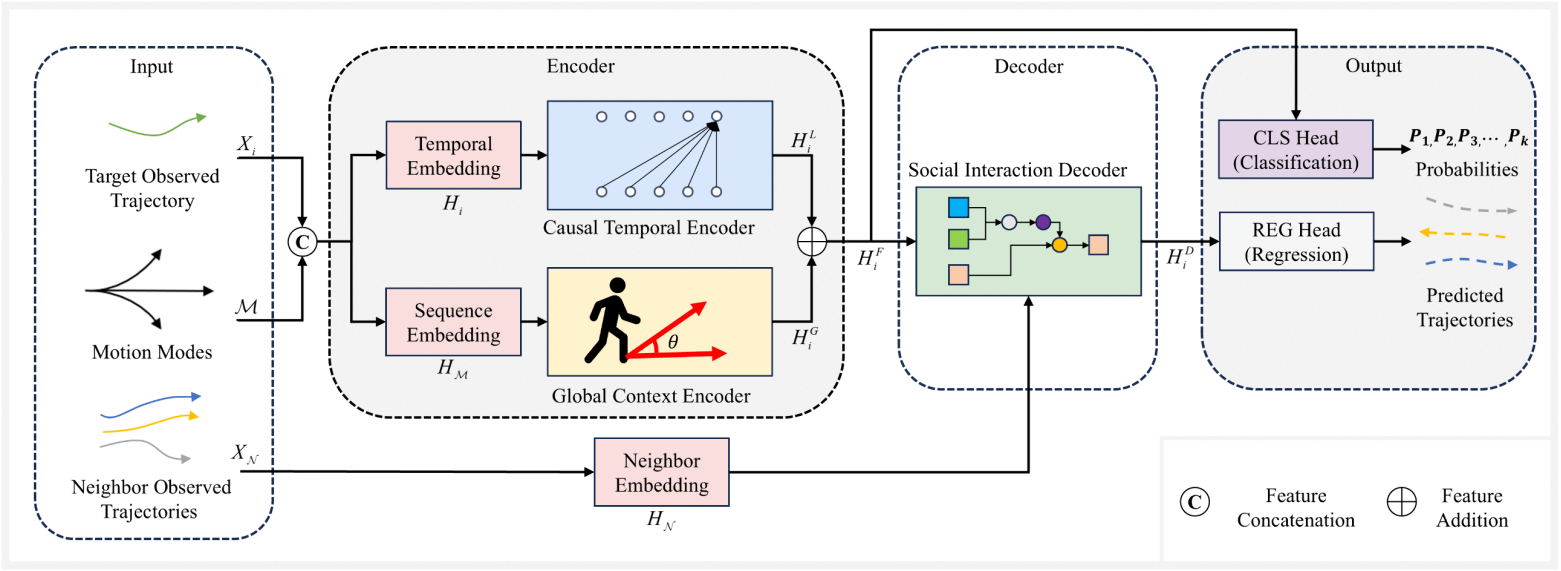
Overview of the proposed LGCMT framework. The target trajectory *X*_*i*_ and neighbor trajectories $\left\{X_{j}\right\}$ are embedded and processed by a local–global collaborative encoder, consisting of a Causal Temporal Encoder (CTE) with SCT-MSA for local dynamics and a Global Context Encoder (GCE) for global motion trends. A motion-pattern library is scored by the CLS head to select top-*K* modes, and a socially-aware non-autoregressive decoder (REG head) generates *K* future trajectory hypotheses in parallel.

Fig 2 illustrates the LGCMT architecture. Input 2D coordinates for the target pedestrian *X*_*i*_ and its neighbors $\left\{X_{j}|j\in {𝒩}_{i}\right\}$ are first embedded into high-dimensional features. The target’s features are then processed by our local-global collaborative encoder (described in the Pedestrian history encoding: A local-global collaborative approach subsection). This encoder has two parallel branches: a Causal Temporal Encoder (CTE) with SCT-MSA to capture local dynamics, and a Global Context Encoder (GCE) with cosine similarity attention for global patterns. Their fused output, $H_{i}^{\text{fused}}$, represents the target’s history. Correspondingly, the historical trajectories of neighbors *X*_*j*_ are processed through a dedicated embedding layer to obtain their feature representations *H*_*j*_ (see the Socially-aware parallel trajectory decoding subsection for details). Next, a pattern scoring module (CLS Head, as detailed in the Structured multi-mode hypothesis generation via motion pattern library subsection) compares $H_{i}^{\text{fused}}$ against a pre-constructed motion pattern library ℳ, selecting the top-*K* motion patterns and their embeddings $\left\{h_{m}^{\text{mode}}\right\}$. Finally, for each selected pattern, a socially-aware non-autoregressive decoding process (detailed in the Socially-aware parallel trajectory decoding subsection) is initiated. It leverages the target’s mode-specific feature representation, incorporates social context $h_{i}^{\text{social}}$ derived from neighbor features *H*_*j*_ via an attention mechanism, and then utilizes a regression head (REG Head) to generate the complete future trajectory *Y*_(*i*,*m*)_ in a single step, outputting *K* trajectory candidates ${𝒴}_{i}$.

### 3.2 Pedestrian history encoding: A local-global collaborative approach

Accurately predicting future pedestrian trajectories hinges on effectively understanding their historical motion. Pedestrian movement is not random; it often comprises a blend of immediate, fine-grained maneuvers and broader, macro-level intentions. To capture this inherent duality, we introduce a local-global collaborative encoder. This encoder processes the observed trajectory $X_{i}=\{p_{\left(i,t\right)}|t=1,\ldots ,T_{\text{obs}}\}$, where $p_{\left(i,t\right)}\in {\mathbb{R}}^{2}$ represents the 2D coordinates of pedestrian *i* at time *t*, and *T*_obs_ is the length of the observation period.

The encoding process starts by considering each of the *N*_lib_ pre-defined motion patterns from the library (see the Structured multi-mode hypothesis generation via motion pattern library subsection). For each pattern *M*_*k*_, a complete candidate sequence *S*_*k*_ is formed by concatenating the observed history *X*_*i*_ with the pattern coordinates *M*_*k*_, spanning the combined observation and prediction horizon ($T=T_{\text{obs}}+T_{\text{pred}}$). These *N*_lib_ candidate sequences are then processed in parallel through two distinct initial embedding pathways, corresponding to the CTE and GCE branches, as illustrated in Fig 2.

For the CTE path, designed to capture local dynamics, each candidate sequence *S*_*k*_ is processed by a Temporal Embedding module. This module transforms each 2D coordinate *p*_*t*_ within *S*_*k*_ into a *d*_model_-dimensional feature vector $h_{t,k}^{0}$:


$$h_{t,k}^{0}=\text{TemporalEmbedding}(p_{t}),\;\text{where }p_{t}\in S_{k},$$ (2)

This yields *N*_lib_ initial embedded sequences $H_{temporal,k}^{0}=h_{t,k}^{0}|t=1,\ldots ,T$, which form the input tensor for the CTE branch.

Concurrently, for the GCE path aiming to capture global context, each candidate sequence *S*_*k*_ is processed by a Sequence Embedding module. This module takes the entire sequence *S*_*k*_ as input and generates a single *d*_model_-dimensional feature vector $h_{global\_embed,k}$ representing the overall context of that specific mode hypothesis:


$$h_{\text{global\_embed},k}=\text{SequenceEmbedding}(S_{k}),$$ (3)

The collection of these *N*_lib_ vectors, forming a tensor $H_{global_{e}mbed}$, serves as the input representation for the GCE branch. These distinct embedding strategies ensure that the subsequent CTE and GCE layers receive input features tailored to their respective tasks of local and global pattern extraction.

#### 3.2.1 Causal temporal encoder (CTE) for efficient local dynamic extraction.

**Fig 3:**
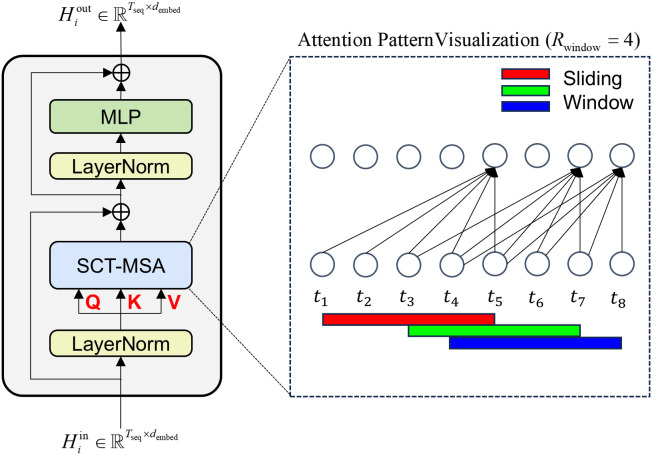
Detailed structure of the Sparse Causal Temporal Multi-head Self-Attention (SCT-MSA) module. The mechanism restricts attention to a local sliding window of size *R*_*window*_ (shaded grey area), ensuring that the feature representation at time *t* depends only on the recent history [*t* − *R*_*window*_, *t*]. This design enforces causali*t*y and reduces computational complexity compared to full self-attention.

The first branch, our CTE, is designed to meticulously capture the fine-grained temporal dynamics from the recent history of a pedestrian’s movement. The cornerstone of the CTE is the SCT-MSA module, illustrated in Fig 3. The design of SCT-MSA intrinsically respects the natural arrow of time in motion by being causal; that is, the feature representation at any time step *t* is influenced exclusively by past and present information ($t^{\prime }\leq t$). Furthermore, SCT-MSA introduces sparsity by confining its attention mechanism to a defined local historical window of size *R*_window_. For each time step *t*, it considers information only from $t^{\prime }\in [\max(1,t-R_{\text{window}}+1),t]$. This focus on localized, recent his*t*ory is crucial for capturing immediate motion cues. The combination of causality and local sparse attention significantly enhances computational efficiency, reducing the self-attention complexity from $𝒪(T_{\text{obs}}^{2})$ per layer, typical of standard Transformers [17], to a more favorable $𝒪(T_{\text{obs}}\cdot R_{\text{window}})$. This makes the CTE well-suited for processing observation sequences where local dependencies are crucial, particularly with large *T*_obs_ and small *R*_window_.

Within each SCT-MSA layer, the input sequence (e.g., $H_{i}^{0}$ for the initial layer) is transformed into Query (Q), Key (K), and Value (V) vectors via distinct linear projections ($W_{q}^{CTE},W_{k}^{CTE},W_{v}^{CTE}$):


$$\left\{ \begin{array}{l} Q_{t}=h_{i,t}^{\text{in}}W_{q}^{CTE},\\ K_{t^{\prime }}=h_{i,t^{\prime }}^{\text{in}}W_{k}^{CTE},\\ V_{t^{\prime }}=h_{i,t^{\prime }}^{\text{in}}W_{v}^{CTE}, \end{array}\right.$$ (4)

where *h*^in^ denotes the feature input to the current layer. Attention scores are then computed using scaled dot-product attention, where a mask, $M_{\text{mask}}^{CTE}$, rigorously enforces both causality and the sparse local window by permitting attention only to positions $t^{\prime }$ within the allowed range:


$${\text{AttentionScores}}^{CTE}(t,t^{\prime })=\frac{Q_{t}(K_{t^{\prime }}{)}^{⊤}}{\sqrt{d_{k}}}+M_{\text{mask}}^{CTE}(t,t^{\prime }),$$ (5)

Here, $d_{k}=d_{\text{model}}/N_{h}$ is the dimension per attention head (*N*_*h*_ heads total). After Softmax normalization, the output feature $h_{\left(i,t\right)}^{\text{out}}$ is a weighted sum of Value vectors from the defined causal sparse window:


$$\begin{array}{cc} h_{i,t}^{\text{out}} & =\sum_{t^{\prime }=\max(1,t-R_{\text{window}}+1)}^{t}\text{Softmax}({\text{AttentionScores}}^{CTE}(t,:){)}_{t^{\prime }}\cdot V_{t^{\prime }}. \end{array}$$ (6)

The SCT-MSA module is then completed with residual connections, Layer Normalization, and a position-wise Feed-Forward Network (FFN), adhering to the standard Transformer block structure [17]. Stacking *L*_*CTE*_ such layers empowers the CTE branch to produce $H_{i}^{\text{local}}$, a feature sequence rich in detailed, short-term motion characteristics.

#### 3.2.2 Global context encoder (GCE) for macro-level patterns.

The second branch of our encoder, the GCE, focuses on discerning broader, macro-level patterns inherent to the target pedestrian’s own movement. It is crucial to clarify that “Global” in this context refers to the temporal global scope of the trajectory sequence, rather than the spatial global scope of the crowd.

Unlike the Social Decoder which handles agent-agent interactions, the GCE is strictly an intra-agent module. It processes the entire candidate sequence (comprising the observed history and a hypothesized future motion mode) as a single input. This holistic view allows the GCE to extract overarching behavioral trends specific to the target’s individual motion intent, independent of social interactions. By isolating the individual’s long-term goal from transient social perturbations, the GCE provides a stable representation of long-term intent.

The GCE consists of *L*_GCE_ Transformer encoder layers [17], distinguished by its use of Cosine Similarity Attention. Query (*Q*_*t*_) and Key ($K_{t^{\prime }}$) vectors are generated as in the CTE. Their similarity, however, is measured using cosine similarity, which emphasizes their directional relationship rather than dot product magnitude, potentially offering a better grasp of overall motion intent:


$$\text{CosineSim}(Q_{t},K_{t^{\prime }})=\frac{Q_{t}\cdot K_{t^{\prime }}}{\|Q_{t}{\|}_{2}\|K_{t^{\prime }}{\|}_{2}}.$$ (7)

Attention scores are derived by scaling this similarity with a learnable parameter γ (an optional mask $M_{\text{mask}}^{\prime }$ is typically unused to allow full global interaction):


$$\begin{array}{cc} \text{AttentionScores}(t,t^{\prime }) & =\gamma \cdot \text{CosineSim}(Q_{t},K_{t^{\prime }})+M_{\text{mask}}^{\prime }(t,t^{\prime }). \end{array}$$ (8)

Following Softmax normalization:


$$\begin{array}{cc} \text{AttentionWeights}(t,t^{\prime }) & =\text{Softmax}{\left(\text{AttentionScores}(t,:)\right)}_{t^{\prime }}. \end{array}$$ (9)

The output feature $h_{\left(i,t\right)}^{\text{out}}$ aggregates information from all historical time steps:


$$h_{i,t}^{\text{out}}=\sum_{t^{\prime }}\text{AttentionWeights}(t,t^{\prime })V_{t^{\prime }}.$$ (10)

Stacking *L*_GCE_ such modified Transformer layers, each incorporating standard Layer Normalization and an FFN, yields $H_{i}^{\text{global}}$, a sequence encoding the global contextual information of the trajectory.

#### 3.2.3 Feature fusion for comprehensive understanding and computational considerations.

Having extracted detailed local dynamics ($H_{i}^{\text{local}}$) with the CTE and broad global patterns ($H_{i}^{\text{global}}$) with the GCE, these complementary representations are integrated to form a holistic understanding of the pedestrian’s historical movement. This is achieved through an element-wise summation:


$$H_{i}^{\text{fused}}=H_{i}^{\text{local}}+H_{i}^{\text{global}}.$$ (11)

This $H_{i}^{\text{fused}}$ serves as the comprehensive historical representation for subsequent model stages.

This dual-branch architecture reflects a deliberate design choice regarding computational complexity. The CTE, with its SCT-MSA module, reduces per-layer attention complexity to approximately $𝒪(T_{\text{obs}}\cdot R_{\text{window}}\cdot d_{\text{model}})$ from the standard $𝒪(T_{\text{obs}}^{2}\cdot d_{\text{model}})$. Its overall complexity (including FFNs of hidden dimension *d*_ff_) is roughly $𝒪(L_{\text{CTE}}\cdot T_{\text{obs}}\cdot (R_{\text{window}}\cdot d_{\text{model}}+d_{\text{model}}\cdot d_{\text{ff}}))$. This renders the CTE highly efficient for long sequences where $R_{\text{window}}\ll T_{\text{obs}}$. Conversely, the GCE maintains $𝒪(T_{\text{obs}}^{2}\cdot d_{\text{model}})$ per-layer attention complexity to capture all-pairs global context, leading to an overall GCE complexity of approximately $𝒪(L_{\text{GCE}}\cdot T_{\text{obs}}\cdot (T_{\text{obs}}\cdot d_{\text{model}}+d_{\text{model}}\cdot d_{\text{ff}}))$. The fusion step adds negligible $𝒪(T_{\text{obs}}\cdot d_{\text{model}})$ complexity. This design allows our model to balance computational load: the CTE efficiently distills local causal dynamic with complexity linear in *T*_obs_ (for fixed *R*_window_), while the GCE, though more intensive, extracts indispensable global context, achieving a synergistic blend of expressive power and efficiency.

### 3.3 Structured multi-mode hypothesis generation via motion pattern library

Pedestrian behavior is inherently multi-mode, meaning individuals often have several plausible future paths. To effectively address this diversity while avoiding the inefficiencies and complex post-processing associated with some traditional generative models, we introduce a structured hypothesis generation approach. This method is centered on a pre-constructed motion pattern library, a collection denoted as $ℳ=\{M_{k}\in {\mathbb{R}}^{T_{\text{pred}}\times 2}|k=1,\ldots ,N_{\text{lib}}\}$. This library comprises *N*_lib_ distinct, typical future motion patterns, each *M*_*k*_ representing a sequence of 2D coordinates over the prediction horizon *T*_pred_.

The creation of this motion pattern library is an offline process performed once using the training dataset. Initially, a large corpus of future trajectory segments, each spanning *T*_pred_ time steps, is collected. To ensure that the learned patterns represent general motion characteristics rather than absolute starting positions, each trajectory segment is normalized, for instance, by translating its initial point to the origin. Following normalization, K-Means clustering, a standard unsupervised learning algorithm, is applied to these trajectory segments. K-Means groups similar trajectories together, and the centroid of each resulting cluster becomes a distinct motion pattern *M*_*k*_ in our library ℳ. The number of patterns, *N*_lib_, is a hyperparameter chosen based on dataset characteristics and desired granularity. Each raw 2D coordinate sequence *M*_*k*_ is then transformed into a learnable *d*_model_-dimensional feature vector, $h_{k}^{\text{mode}}$, using an embedding layer denoted ${\text{MLP}}_{\text{mode\_embed}}$. This allows the model to work with richer representations of these patterns.

During inference, the model utilizes the fused feature tensor $H_{i}^{\text{fused}}$ obtained after the local-global feature fusion step (see the Feature fusion for comprehensive understanding and computational considerations subsubsection). This tensor, with dimensions reflecting the batch size, the *N*_lib_ mode hypotheses, and the feature dimension (*B* × *N*_lib_ × *d*_model_), encapsulates the comprehensive representation for each potential future scenario. This entire tensor $H_{i}^{\text{fused}}$ is then directly fed into the scoring network, MLP_score_ (referred to as the CLS Head in Fig 2). The scoring network, implemented as a linear layer mapping from *d*_model_ to 1, operates independently on the feature vector corresponding to each of the *N*_lib_ mode hypotheses:


$${\text{Scores}}_{i}={\text{MLP}}_{\text{score}}(H_{i}^{\text{fused}}),$$ (12)

where Scores_*i*_ is now understood as a tensor of shape *B* × *N*_lib_, containing the calculated score for each of the *N*_lib_ patterns based on their respective fused representations.

These scores are converted into a probability distribution *p*_*i*_ over the patterns via the Softmax function, where *p*_(*i*,*k*)_ indicates the likelihood of pattern *M*_*k*_ for pedestrian *i*:


$$p_{i}=\text{Softmax}({\text{Scores}}_{i})\in {\mathbb{R}}^{N_{\text{lib}}},$$ (13)

For training, target modes and probabilities guide the learning of *L*_pred_ and *L*_mode_ (detailed in the Training strategy and loss functions subsection). In inference, the top-*K* patterns, identified by $\text{TopK}(p_{i})$, and their embeddings $\left\{h_{m}^{\text{mode}}\right\}$ direct the parallel generation of multiple trajectory hypotheses.

This library-based mechanism enhances prediction quality and interpretability by incorporating explicit prior knowledge of common behaviors, guiding generation towards plausible outcomes. While the library’s coverage limits its ability to represent entirely novel behaviors and discretizing motion might lose some nuances, its careful construction is expected to significantly improve the generation of diverse and realistic trajectory candidates. The quality and representativeness of the library are key considerations.

### 3.4 Socially-aware parallel trajectory decoding

Effective trajectory prediction requires not only understanding an individual’s past movement and intended goals but also their dynamic interactions with surrounding individuals. The Socially-Aware Parallel Trajectory Decoding approach proposed in this paper addresses this by simultaneously generating multiple future path hypotheses for a target pedestrian, each explicitly conditioned on the dynamic social context. This capability is vital for creating realistic and reliable predictions, particularly in scenarios with complex pedestrian interplay where movements are heavily interdependent.

To incorporate these social influences efficiently, the decoder utilizes the neighbor set ${𝒩}_{i}$ identified via the spatial distance threshold τ (as defined in Section 3.1). This design is critical for scalability. Let *P* denote the total number of pedestrians in the scene and *N*_max_ be the maximum number of neighbors considered (set to 50 in our experiments). While global attention mechanisms inherently suffer from quadratic complexity $𝒪(P^{2}\cdot d_{\text{model}})$, our distance-based filtering reduces the interaction scope to a local subset. Consequently, the computational cost for the social module scales linearly $𝒪(P\cdot N_{\text{max}}\cdot d_{\text{model}})$. This ensures that the model remains lightweight and responsive even in high-density crowds. The historical trajectory *X*_*j*_ of each neighbor $j\in {𝒩}_{i}$ is processed to obtain its summarized context vector *c*_*j*_. In our proposed LGCMT model, this is achieved by first flattening its historical trajectory *X*_*j*_ into a single vector, which is then processed through a dedicated linear embedding layer. This approach provides computationally efficient contextual representations *c*_*j*_ for each neighboring agent, suitable for the subsequent social attention module.

For each of the *K* motion patterns selected from the library (as described in the Structured multi-mode hypothesis generation via motion pattern library subsection), the decoding process generates a corresponding future trajectory *Y*_(*i*,*m*)_. This generation process is non-autoregressive, predicting all *T*_pred_ future coordinates simultaneously for enhanced inference speed. The process begins with a feature representation for the target pedestrian *i* that is specific to the selected mode *m*. This feature, let’s denote it as $h_{\left(i,m\right)}^{\text{pre-social}}$, is derived from the target’s fused historical features $H_{i}^{\text{fused}}$ and incorporates the corresponding pattern embedding $h_{m}^{\text{mode}}$.

Crucially, social context is then integrated in a mode-specific manner using a social attention mechanism. This mechanism takes the target pedestrian’s mode-specific feature $h_{\left(i,m\right)}^{\text{pre-social}}$ as the query, while the set of neighbor context vectors $\left\{c_{j}|j\in {𝒩}_{i}\right\}$ serve as keys and values. This allows the model to dynamically weigh the influence of each surrounding individual conditioned specifically on the motion hypothesis *m* being considered:


$$\begin{array}{cc} {\tilde{h}}_{i,m}^{\text{social}} & =\text{SocialAttention}(\text{query}=h_{i,m}^{\text{pre-social}},\\ & \;\text{keys}=\{c_{j}|j\in {𝒩}_{i}\},\text{values}=\{c_{j}|j\in {𝒩}_{i}\}). \end{array}$$ (14)

This yields a socially-informed, mode-specific feature vector, ${\tilde{h}}_{\left(i,m\right)}^{\text{social}}$, which encapsulates the target’s history, the specific motion pattern’s influence, and relevant social interactions pertinent to that mode.

This resultant feature ${\tilde{h}}_{\left(i,m\right)}^{\text{social}}$ is then fed directly into a shared regression network, MLP_reg_. This network outputs the complete *T*_pred_-step future trajectory *Y*_(*i*,*m*)_ for the corresponding mode *m*:


$$Y_{i,m}={\text{MLP}}_{\text{reg}}({\tilde{h}}_{i,m}^{\text{social}})\in {\mathbb{R}}^{T_{\text{pred}}\times 2}.$$ (15)

This entire procedure, from preparing the mode-specific query to final regression, is executed in parallel for each of the *K* selected modes, efficiently producing the diverse set of ${𝒴}_{i}$ trajectory candidates.

This parallel and non-autoregressive design efficiently produces a diverse set of ${𝒴}_{i}$ trajectory candidates. By bypassing the iterative recurrence of traditional LSTM-based decoders (which require *T*_pred_ sequential steps), our regression head generates the full prediction horizon in a single forward pass (𝒪(1) temporal complexity). Combined with the linear spatial complexity of the social attention, this architecture achieves a significant reduction in inference latency. The explicit modeling of social interactions makes this decoding strategy particularly effective in complex real-world environments, while the optimized computational design ensures adaptability across varying crowd densities.

**Algorithm 1: LGCMT Prediction Process**



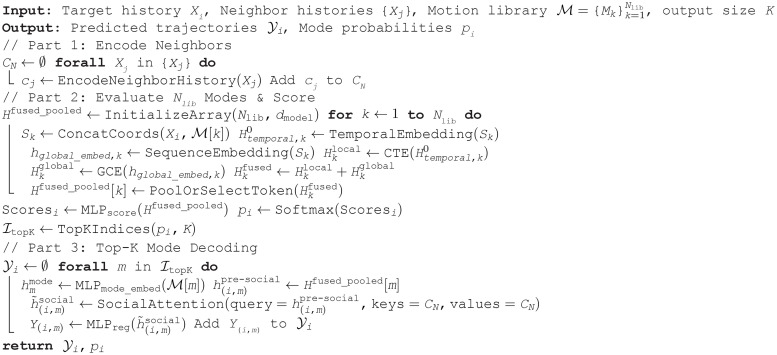



### 3.5 Training strategy and loss functions

The model is trained by minimizing a composite loss function, *L*_total_, designed to ensure both prediction accuracy and appropriate mode selection. This total loss is a weighted sum of a trajectory prediction loss *L*_pred_ and a mode classification loss *L*_mode_, balanced by hyperparameters $\lambda_{\text{pred}}$ and $\lambda_{\text{mode}}$:


$$L_{\text{total}}=\lambda_{\text{pred}}L_{\text{pred}}+\lambda_{\text{mode}}L_{\text{mode}}.$$ (16)

The trajectory prediction loss, *L*_pred_, is formulated to guide the model towards accurate trajectory generation conditioned on the most suitable motion pattern during the training phase. Specifically, for each ground truth future trajectory $Y_{i}^{\text{GT}}$, we first identify the index m^*^ of the motion pattern $M_{m^{*}}$ within the library ℳ that exhibits the highest similarity to $Y_{i}^{\text{GT}}$. The model is then explicitly trained to produce a single trajectory prediction, $Y_{\left(i,m^{*}\right)}$, by utilizing only the computational path associated with this best-matching mode m^*^. The prediction loss *L*_pred_ is subsequently computed as the Smooth L1 loss between this specific prediction $Y_{\left(i,m^{*}\right)}$ and the ground truth $Y_{i}^{\text{GT}}$:


$$L_{\text{pred}}=\text{SmoothL1}(Y_{i,m^{*}},Y_{i}^{\text{GT}}),$$ (17)

where SmoothL1(·,·) represents the Smooth L1 loss function, typically averaged over all predicted time steps and samples. This training approach ensures that the learning signal focuses on generating accurate predictions from the identified ‘target’ mode, differing fundamentally from the inference-time evaluation procedure where the best among *k*_eval_ generated hypotheses is selected based on distance metrics.

The mode classification loss, *L*_mode_, guides the model to identify appropriate underlying motion patterns from the library. A target “soft” probability distribution *q*_*i*_ over the *N*_lib_ library patterns is first derived by comparing the normalized ground truth future $Y_{\left(i,\text{norm}\right)}^{\text{GT}}$ to each normalized library pattern *M*_*k*_. Similarity scores *s*_(*i*,*k*)_ are converted to probabilities *q*_(*i*,*k*)_ using a Softmax function with temperature τ:


$$q_{i,k}=\frac{\exp(s_{i,k}/\tau )}{\sum_{j=1}^{N_{\text{lib}}}\exp(s_{i,j}/\tau )}.$$ (18)

*L*_mode_ is then the cross-entropy between the model’s predicted mode distribution *p*_*i*_ (shown in Eq 13) and this target *q*_*i*_:


$$L_{\text{mode}}=-\sum_{k=1}^{N_{\text{lib}}}q_{i,k}\log(p_{i,k}).$$ (19)

Jointly optimizing *L*_pred_ and *L*_mode_ promotes high-quality, diverse, and contextually appropriate predictions. Algorithm 1 summarizes the complete forward pass of our proposed model.

## 4 Results

### 4.1 Experimental setup

#### 4.1.1 Datasets.

We evaluate the LGCMT model on the widely used ETH [25] and UCY [26] benchmarks. These benchmarks comprise five distinct scenes, namely ETH, HOTEL, UNIV, ZARA1, and ZARA2, featuring varied pedestrian densities and interaction patterns. The data consists of 2D coordinates recorded at 2.5 Hz. We adhere to standard evaluation protocols [8,10], observing trajectories for 8 time steps (corresponding to 3.2 seconds, denoted *T*_obs_) and predicting the subsequent 12 steps (covering 4.8 seconds, denoted *T*_pred_). A Leave-One-Out Cross-Validation (LOOCV) strategy is employed for evaluation, where the model is trained on four scenes and tested on the remaining fifth scene, iterating this process so that each scene serves as the test set once. Input trajectory observations undergo normalization: the starting point is translated to the origin, and the trajectory is rotated to align its initial motion direction approximately with the X-axis.

In addition, we evaluate our model on the Stanford Drone Dataset (SDD) [27], a large-scale benchmark with higher crowd density and scene complexity. For fair comparison, we adopt the same *T*_obs_ = 8 and *T*_pred_ = 12 settings.

#### 4.1.2 Evaluation metrics.

Model performance is quantified using the Average Displacement Error (ADE) and Final Displacement Error (FDE). ADE measures the mean L2 distance between the predicted trajectory ${\hat{Y}}_{i}$ and the ground truth trajectory $Y_{i}^{\text{GT}}$ over all predicted time steps:


$${\text{ADE}}_{i}=\frac{1}{T_{\text{pred}}}\sum_{t=T_{\text{obs}}+1}^{T_{\text{obs}}+T_{\text{pred}}}{\left\|{\hat{p}}_{i,t}-p_{i,t}^{\text{GT}}\right\|}_{2}.$$ (20)

FDE calculates the L2 distance specifically at the final predicted time step *T*_pred_:


$${\text{FDE}}_{i}={\left\|{\hat{p}}_{i,T_{\text{obs}}+T_{\text{pred}}}-p_{i,T_{\text{obs}}+T_{\text{pred}}}^{\text{GT}}\right\|}_{2}.$$ (21)

To account for the inherent multi-modality of future trajectories, we follow common practice [5] by generating *K* potential trajectories and reporting the minimum ADE and FDE among these candidates, denoted minADE_*K*_ and minFDE_*K*_, averaged across all test samples. Unless otherwise specified, we use *K* = 20. Lower ADE and FDE values indicate better prediction accuracy.

#### 4.1.3 Hyperparameter settings.

The proposed LGCMT model was implemented using the PyTorch framework, and all experiments were conducted on an NVIDIA RTX 4070 GPU. The core motion mode library is constructed offline via K-Means clustering. The size of the motion library *N*_lib_ was optimized per scene: 50 for ETH, 90 for HOTEL, 50 for UNIV, 70 for ZARA1, and 50 for ZARA2, while *N*_lib_ = 100 was used for the SDD. During inference, we select the top *K* = 20 modes, consistent with the evaluation protocol. Regarding the neighbor selection strategy defined in Section 3.1, we set the spatial distance threshold τ to 2.0 meters for the ETH/UCY datasets and 5.0 units for the SDD to capture socially significant interactions within the respective spatial scales.

Regarding model architecture, the core hidden dimension *d*_model_ was set to 256 for the ETH and HOTEL datasets, 128 for the UNIV, ZARA1, and ZARA2 datasets, and 64 for the SDD. The local-global collaborative encoder employs 3 stacked Transformer blocks for the ETH dataset and 2 blocks for all other datasets (including SDD). All multi-head attention mechanisms within the encoders and decoders utilize 4 attention heads. The local history window size *R*_window_ for the SCT-MSA module was also tuned specifically for each scene: 4 for ETH and HOTEL, 7 for UNIV and ZARA1, 5 for ZARA2, and 3 for the SDD. For training, we used a batch size of 128 and adhered to the optimization strategy detailed in the Training strategy and loss functions subsection.

### 4.2 Comparison with existing methods

To rigorously evaluate the proposed LGCMT framework, we compare it against a comprehensive set of contemporary methods detailed in Table 1. These include Social-STGCNN [16], STAR [6], PECNet [28], AgentFormer [5], SGCN [15], SIT [29], MemoNet [30], SocialVAE [21], TUTR [24], BCDiff [7], FlowChain [23], SMEMO [4], Social NSTransformers [32], TP-EGT [18], TPPO [31], Social Entropy Informer [33], Social Informer [34], and W-DGTrans [35].

**Table 1:** Performance comparison on the ETH and UCY datasets. Values represent minADE/minFDE in meters. The ↓ symbol indicates that lower values are better. The best results are shown in bold.

Model	Year	ETH ↓	HOTEL ↓	UNIV ↓	ZARA1 ↓	ZARA2 ↓	AVG ↓
Social-STGCNN [16]	2020	0.64/1.11	0.49/0.85	0.44/0.79	0.34/0.53	0.30/0.48	0.44/0.75
STAR [6]	2020	0.36/0.64	0.17/0.36	0.31/0.62	0.29/0.52	0.22/0.46	0.26/0.53
PECNet [28]	2020	0.54/0.87	0.18/0.24	0.35/0.60	0.22/0.39	0.17/0.30	0.29/0.48
AgentFormer [5]	2021	0.45/0.75	0.14/0.22	0.25/0.46	0.18/0.30	0.14/**0.24**	0.23/0.39
SGCN [15]	2021	0.52/1.03	0.32/0.55	0.37/0.70	0.29/0.53	0.25/0.45	0.37/0.65
SIT [29]	2022	0.39/0.62	0.14/0.22	0.27/0.47	0.19/0.33	0.16/0.29	0.23/0.38
MemoNet [30]	2022	0.40/0.61	**0.11/0.17**	0.24/0.43	0.18/0.32	0.14/**0.24**	0.21/0.35
SocialVAE [21]	2022	0.47/0.76	0.14/0.22	0.25/0.47	0.20/0.37	0.14/0.28	0.24/0.42
TUTR [24]	2023	0.40/0.61	**0.11**/0.18	**0.23**/0.42	0.18/0.34	**0.13**/0.25	0.21/0.36
BCDiff [7]	2023	0.53/0.91	0.17/0.27	0.24/0.40	0.21/0.37	0.16/0.26	0.26/0.44
FlowChain [23]	2023	0.55/0.99	0.20/0.35	0.29/0.54	0.22/0.40	0.20/0.34	0.29/0.52
SMEMO [4]	2024	0.39/0.59	0.14/0.20	**0.23/0.41**	0.19/0.32	0.15/0.26	0.22/0.35
TP-EGT [18]	2024	0.41/0.68	0.13/0.21	0.29/0.50	0.18/0.30	0.16/0.27	0.23/0.39
TPPO [31]	2024	0.75/1.27	0.36/0.70	0.39/0.74	0.22/0.37	0.23/0.45	0.39/0.71
Social NSTransformers [32]	2024	0.40/0.71	0.29/0.47	0.39/0.73	0.34/0.62	0.31/0.57	0.35/0.62
Social Entropy Informer [33]	2025	0.34/0.64	0.19/0.33	0.29/0.61	0.24/0.52	0.22/0.46	0.26/0.51
Social Informer [34]	2026	0.34/0.61	0.17/0.33	0.30/0.63	0.24/0.52	0.21/0.44	0.25/0.51
W-DGTrans [35]	2026	**0.28**/0.56	0.17/0.29	0.25/**0.41**	**0.16/0.25**	0.17/0.27	0.21/0.36
LGCMT (Ours)	—	0.36/**0.55**	**0.11/0.17**	**0.23**/0.42	0.18/0.34	0.14/**0.24**	**0.20/0.34**

#### 4.2.1 Performance on ETH/UCY datasets.

On the standard ETH and UCY benchmarks, LGCMT demonstrates robust performance, achieving the lowest average errors across all compared methods with a minADE of 0.20 and a minFDE of 0.34. This represents a substantial improvement over earlier baselines and a competitive edge over the most recent approaches.

Specifically, we compare our method against the recent works of TP-EGT [18] and TPPO [31] as highlighted in recent literature. LGCMT outperforms the graph-transformer-based TP-EGT (Average ADE 0.23) by approximately 13.0% and significantly surpasses the pose-optimization-based TPPO (Average ADE 0.39) with a reduction in error of roughly 48.7%. Furthermore, compared to the 2026 baseline W-DGTrans [35], which reports an average ADE of 0.21 meters, our model maintains a performance advantage, particularly in the HOTEL and ZARA2 scenes where distinct motion patterns and social interactions are prevalent.

The breakdown by scene reveals that LGCMT achieves the best reported ADE in the HOTEL (0.11), UNIV (0.23), and ZARA2 (0.14) subsets. This consistency across datasets with varying pedestrian densities validates the effectiveness of the local-global collaborative encoder. By simultaneously capturing the fine-grained local dynamics and the long-term individual intent, the model effectively mitigates the trade-off often observed in other methods that may overfit to specific scene types.

#### 4.2.2 Performance on SDD dataset.

To address the limitations associated with the relatively small scale of the ETH/UCY datasets and to test the model’s scalability in high-density, real-world environments, we extended our evaluation to the SDD [27]. As shown in Table 2, SDD presents significantly greater challenges due to its bird’s-eye view, diverse agent types (including cyclists and skaters), and complex static obstacles.

**Table 2:** Performance comparison on the stanford drone dataset (SDD). Prediction errors are reported as ADE/FDE in pixels. Values are averaged over the best of 20 predicted trajectories. Lower values are better.

Model	Year	SDD ADE ↓	SDD FDE ↓
PECNet [28]	2020	9.29	15.93
MemoNet [30]	2022	8.56	**12.66**
SocialVAE [21]	2022	8.88	14.81
SIT [29]	2022	9.13	15.42
BCDiff [7]	2023	9.05	14.86
TUTR [24]	2023	7.99	13.38
SMEMO [4]	2024	8.11	13.06
Social NSTransformers [32]	2024	10.92	18.01
Social Entropy Informer [33]	2025	8.72	13.35
LGCMT	—	**7.90**	13.04

In this rigorous benchmark, LGCMT achieves a minADE of 7.90 pixels and a minFDE of 13.04 pixels. These results surpass those of competitive baselines, including TUTR [24] (7.99 pixels ADE) and SMEMO [4] (8.11 pixels ADE). The superior performance on SDD is particularly noteworthy as it confirms that the proposed spatial neighbor filtering strategy allows the model to scale efficiently to crowded scenes. Unlike global attention mechanisms that may suffer from noise accumulation when processing dozens of agents, our method maintains precision by focusing on socially relevant neighbors, thereby demonstrating strong robustness and generalization capabilities in complex, unstructured environments.

### 4.3 Ablation study

To rigorously validate the architectural choices underpinning the LGCMT model and quantify their individual contributions, we conducted comprehensive ablation studies on the ETH/UCY datasets. These experiments assessed the impact of removing or altering key components on both predictive accuracy, measured by average minimum ADE and FDE over 20 samples in meters, and computational efficiency via average inference time in milliseconds. All ablation experiments maintained the primary experimental setup on an NVIDIA RTX 4070 GPU.

#### 4.3.1 Component effectiveness analysis.

We first investigated the contribution of LGCMT’s primary architectural modules by systematically removing or modifying them. The results, detailed in Table 3, reveal the significance of each design decision.

**Table 3:** Ablation study results on the ETH and UCY datasets. All values are minADE/minFDE in meters. The performance of the full model is highlighted in bold.

Method	ETH	HOTEL	UNIV	ZARA1	ZARA2	AVG
Full LGCMT	**0.36/0.55**	**0.11/0.17**	**0.23/0.42**	**0.18/0.34**	**0.14/0.24**	**0.20/0.34**
LGCMT w/o Global	0.42/0.63	0.14/0.23	0.25/0.48	0.21/0.39	0.16/0.30	0.24/0.41
LGCMT w/o Local	0.41/0.62	0.12/0.19	0.24/0.45	0.19/0.36	0.15/0.27	0.22/0.38
LGCMT w/o Social	0.44/0.70	0.18/0.30	0.29/0.55	0.28/0.52	0.22/0.41	0.28/0.50
LGCMT w/o Modes	0.50/0.80	0.20/0.32	0.35/0.65	0.30/0.58	0.25/0.50	0.32/0.58
LGCMT w. Std Attn (Local)	0.38/0.60	0.13/0.19	0.23/0.43	0.19/0.35	0.14/0.26	0.21/0.37
LGCMT w. Std Attn (Global)	0.39/0.58	0.12/0.20	0.24/0.43	0.20/0.37	0.15/0.27	0.22/0.37

The necessity of the local-global collaborative encoding strategy is immediately apparent. Removing the global context encoder, hereafter referred to as GCE, led to a substantial performance decline; average ADE increased by 20.0% to 0.24 and average FDE rose by 20.6% to 0.41. This underscores the critical role of the GCE in capturing broader trajectory trends and inferring longer-term pedestrian intent. Similarly, ablating the causal temporal encoder, hereafter CTE, also hampered predictive accuracy, elevating average ADE by 10.0% to 0.22 and FDE by 11.8% to 0.38. While this impact was less severe than removing the global context, it confirms the importance of the CTE’s fine-grained local motion modeling. These findings collectively indicate that relying solely on a single temporal scale is insufficient; LGCMT’s strength derives from synergistically integrating local dynamics captured by the CTE with global motion understanding provided by the GCE.

The experiments further highlight the profound impact of the prediction guidance mechanism. The most significant performance degradation occurred when the motion mode library was removed. Without this guidance, average ADE surged by 60.0% to 0.32, and FDE increased dramatically by 70.6% to 0.58. This starkly illustrates that generating structured multi-mode hypotheses, informed by learned motion patterns, is fundamental to the model’s accuracy and its capacity for diverse, plausible predictions. Additionally, neglecting social interactions proved detrimental. Removing social context modeling resulted in a 40.0% increase in average ADE to 0.28 and a 47.1% increase in average FDE to 0.50, confirming that accounting for the influence of nearby pedestrians remains crucial for realistic trajectory forecasting, particularly in interactive settings.

Finally, the specific choice of attention mechanisms within the encoders was validated. Replacing the specialized Sparse Causal Temporal Multi-head Self-Attention, known as SCT-MSA, in the CTE with standard self-attention resulted in a noticeable drop in performance, yielding an average ADE/FDE of 0.21/0.37. A similar decline to 0.22/0.37 was observed when the GCE’s cosine similarity attention was substituted with standard dot-product attention. These outcomes affirm that the tailored designs of SCT-MSA for local temporal dependencies and cosine similarity attention for global directional trends are more effective within their respective LGCMT modules than generic attention approaches.

#### 4.3.2 Complexity and inference speed analysis.

Beyond predictive accuracy, the practical utility of a forecasting model relies heavily on its computational efficiency. To comprehensively evaluate the real-time performance of the algorithm, we analyzed three key indicators: model parameters (Params), computational complexity (FLOPs), and actual Inference Time.

To ensure a fair and rigorous comparison, we established a unified evaluation protocol. All models listed in Table 4 were re-evaluated on a workstation equipped with an NVIDIA GeForce RTX 4070 GPU. We strictly followed the standard real-time latency evaluation criteria: the inference time was measured with a batch size of 1 and a sampling number of *K* = 20. Furthermore, all reported data are the average of measurements taken after a warm-up period to exclude system initialization noise and random fluctuations. The values are averaged across the five ETH/UCY datasets to mitigate biases from specific scene characteristics.

**Table 4:** Comparison of Model Complexity and Inference Speed. Params are reported in millions (M), FLOPs in gigaflops (G), and inference time in milliseconds (ms). All models were evaluated on an NVIDIA RTX 4070 GPU.

Method	Params (M)	FLOPs (G)	Inference Time (ms)
Social-STGCNN	0.008	0.002	1.59
STAR	0.965	0.017	26.36
AgentFormer	6.780	3.080	46.58
MemoNet	5.280	0.408	156.96
SocialVAE^†^	1.540	0.150	16.27
TUTR	0.440	0.065	1.89
LGCMT (Ours)	0.679	0.086	2.79

^†^ SocialVAE is evaluated without FPC (Fitted Posterior Check) to ensure fair real-time comparison.

It is worth noting that the inference time for the same algorithm varies across different scenarios. This variance is primarily attributed to crowd density. Most interaction-aware models, including LGCMT, employ mechanisms where the computational cost scales with the number of agents in the frame. Consequently, densely populated scenarios (such as UNIV) naturally incur slightly higher latency compared to sparse scenarios (such as ETH).

As shown in Table 4, LGCMT achieves an optimal balance between accuracy and efficiency (2.79 ms). When compared to complex Transformer-based architectures, our model demonstrates a significant speed advantage. For instance, MemoNet (156.96 ms) involves processing continuous features alongside high-overhead retrieval operations from an external memory bank, which inherently limits its inference speed. Similarly, AgentFormer (46.58 ms) requires heavy computation for its dense attention mechanisms. Regarding SocialVAE, while it achieves competitive speed (16.27 ms), this metric is recorded without its “Fitted Posterior Check (FPC).” Although FPC can improve precision, it increases latency to approximately 2.8 seconds per scene, rendering it unsuitable for real-time applications. While lightweight baselines like Social-STGCNN (1.59 ms) and TUTR (1.89 ms) are marginally faster, LGCMT maintains a comparable millisecond-level speed while offering more robust trajectory modeling capabilities. This analysis confirms that LGCMT is well-suited for deployment in dynamic environments where both accuracy and low latency are critical.

**Impact of Hidden Dimensions:** To further explore the scalability and the trade-off between model capacity and efficiency, we conducted an ablation study on the SDD dataset by varying the hidden dimension size (H∈{16,64,128,256}). As detailed in Table 5, increasing the hidden dimension from 16 to 256 leads to a substantial increase in computational cost: parameters grow from 0.08 M to 2.47 M, and FLOPs double from 0.13 G to 0.26 G. However, this increase in complexity does not strictly correlate with performance gains. The model achieves its best predictive accuracy (ADE = 7.90) at *H* = 64, with an inference time of just 3.02 ms. Interestingly, larger dimensions result in slight performance degradation (ADE = 8.12), likely due to overfitting on the trajectory data. Conversely, an extremely small dimension (*H* = 16) limits the model’s representational power, leading to higher errors. Consequently, we selected *H* = 64 as the optimal configuration for our main experiments, as it minimizes computational overhead while maximizing prediction accuracy.

**Table 5:** Impact of hidden dimensions on model performance on the SDD dataset.

Hidden Dimension	Params (M)	FLOPs (G)	Time (ms)	ADE	FDE
256	2.47	0.26	3.65	8.12	13.40
128	0.68	0.17	3.45	8.11	13.41
64	0.23	0.14	3.02	7.90	13.04
16	0.08	0.13	2.91	8.17	13.41

#### 4.3.3 Robustness analysis.

To verify the stability and reproducibility of LGCMT, we conducted repeated experiments using 5 different random seeds on both the ETH/UCY and SDD datasets. Table 6 reports the statistics in the format of Mean ± Standard Deviation (Std). While the main results in Table 1 report the best-performing model to ensure a fair comparison with baselines, the results here demonstrate that the deviation between the mean performance and the best run is minimal. For instance, on the challenging ETH scene, the mean ADE is 0.37m compared to the best run of 0.36m. The extremely low standard deviations (e.g., ±0.001 on HOTEL and UNIV) confirm that LGCMT is robust to initialization and achieves consistent performance.

**Table 6:** Robustness analysis. We report the Mean ± Standard Deviation over 5 independent runs. The Mean is reported to 2 decimal places, and the Standard Deviation to 3 decimal places to highlight the minimal variance.

Dataset	ADE (Mean ± Std)	FDE (Mean ± Std)
ETH	0.37 ± 0.008	0.56 ± 0.023
HOTEL	0.11 ± 0.001	0.17 ± 0.001
UNIV	0.23 ± 0.001	0.42 ± 0.001
ZARA1	0.18 ± 0.001	0.34 ± 0.001
ZARA2	0.14 ± 0.002	0.25 ± 0.003
SDD	7.93 ± 0.026	13.11 ± 0.056

#### 4.3.4 Impact of non-autoregressive decoding.

To isolate the contribution of our chosen decoding strategy, we explicitly compared the standard non-autoregressive (NAR) LGCMT against an autoregressive (AR) counterpart. This baseline, termed LGCMT (AR), utilized the identical encoder architecture but employed a traditional sequential decoder. Table 7 presents the comparison regarding both predictive accuracy, ADE/FDE, and inference time.

**Table 7:** Comparison of autoregressive (AR) and non-autoregressive (NAR) versions of LGCMT. Predictive performance is measured in ADE/FDE (meters), and inference time is in milliseconds (ms). Results for the superior NAR model are in bold.

Method	ETH	HOTEL	UNIV	ZARA1	ZARA2	AVG
*Predictive Performance (ADE/FDE)*						
LGCMT (AR)	0.44/0.65	0.19/0.26	0.29/0.45	0.25/0.41	0.18/0.30	0.27/0.41
LGCMT (NAR)	**0.36/0.55**	**0.11/0.17**	**0.23/0.42**	**0.18/0.34**	**0.14/0.24**	**0.20/0.34**
*Inference Time (ms)*						
LGCMT (AR)	81.91	83.44	131.58	80.62	87.20	87.55
LGCMT (NAR)	**2.73**	**2.64**	**3.07**	**2.69**	**2.81**	**2.79**

The advantages of the NAR approach are clear. Regarding predictive accuracy, the NAR model significantly outperformed its AR variant. Average ADE decreased by 25.9% from 0.27 to 0.20, and average FDE saw a 17.1% reduction from 0.41 to 0.34. This accuracy enhancement likely arises from the NAR mechanism generating the full sequence concurrently, mitigating the error accumulation often problematic in step-by-step autoregressive predictions.

The efficiency benefits are even more striking. The NAR-based LGCMT required only 2.79 milliseconds for inference on average. This is approximately 31 times faster than the 87.55 milliseconds needed by the LGCMT (AR) model. This dramatic speed-up is a direct result of the NAR decoder’s parallel computation across all future time steps, contrasting sharply with the inherent sequential processing of AR decoding. This finding strongly advocates for the NAR strategy in applications demanding both high accuracy and rapid response times.

#### 4.3.5 Key hyperparameter sensitivity analysis.

We further investigated how LGCMT’s performance responds to variations in two key hyperparameters: the motion mode library size *N*_lib_, and the local history window size *R*_window_ used within the SCT-MSA module. Our primary results employed scene-specific optimal values for *N*_lib_, ranging from 50 to 90, and for *R*_window_, ranging from 4 to 7, achieving the benchmark 0.20/0.34 average ADE/FDE. The following analysis explores performance when these hyperparameters are set uniformly across all scenes.

**Fig 4:**
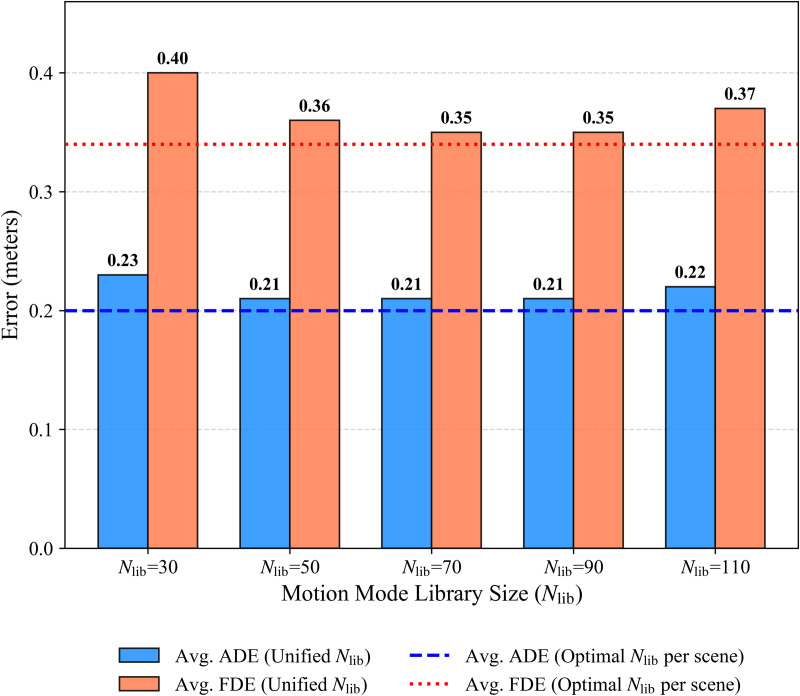
Sensitivity to the motion-pattern library size *N*_lib_. Bars report the average minADE/minFDE when a single *N*_lib_ is used for all scenes. Horizontal dashed lines denote the baseline obtained with scene-specific optimal *N*_lib_. Performance is stable for $N_{\text{lib}}\in [50,90]$, while per-scene tuning achieves the best accuracy.

First, varying the motion mode library size *N*_lib_ uniformly across values {30, 50, 70, 90, 110} revealed performance trends depicted in Fig 4. A small library size where *N*_lib_ equals 30 yielded a noticeable drop to 0.23/0.40 average ADE/FDE, suggesting insufficient capacity to capture motion diversity. Performance stabilized around 0.21 average ADE and 0.35–0.36 average FDE for *N*_lib_ between 50 and 90. Increasing *N*_lib_ further to 110 resulted in a slight degradation to 0.22/0.37. This indicates that while a sufficiently large library is crucial, an excessively large *N*_lib_ offers diminishing returns and can slightly dilute performance, reinforcing the benefit of scene-specific tuning.

**Fig 5:**
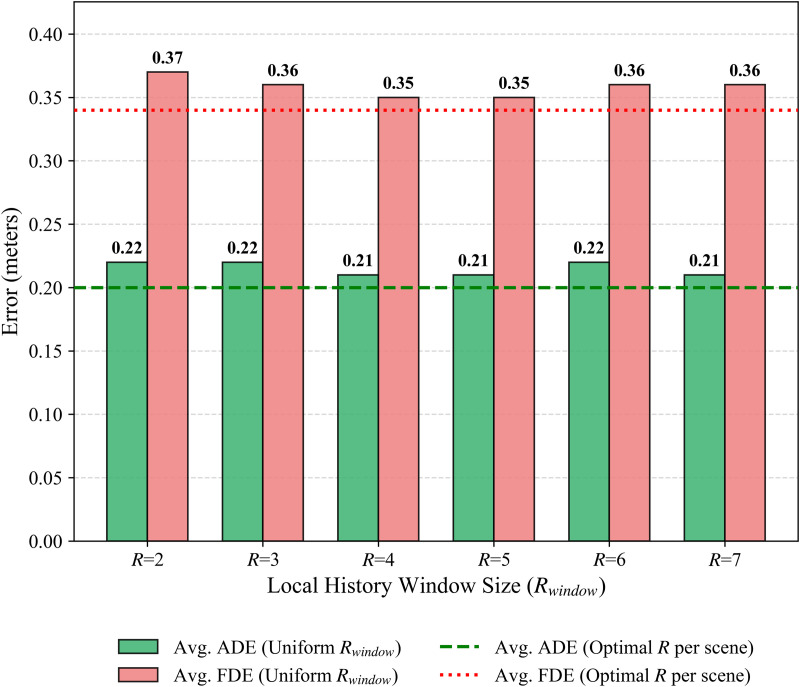
Sensitivity to the local history window size *R*_window_ in SCT-MSA. Bars report the average minADE/minFDE when a single $R_{\text{window}}\in [2,7]$ is used for all scenes. Horizontal dashed lines denote the baseline obtained with scene-specific optimal *R*_window_. The model is robust to *R*_window_, with stable performance around *R*_window_ = 4–5.

Next, evaluating the influence of the local history window size *R*_window_ with uniform values from {2, 3, 4, 5, 6, 7}, as shown in Fig 5, indicated greater robustness compared to variations in *N*_lib_. Across the tested range, average ADE remained between 0.21 and 0.22, and average FDE between 0.35 and 0.37. Settings such as *R*_window_ = 4 or *R*_window_ = 5 produced a solid 0.21/0.35 average ADE/FDE. Even extreme values did not cause sharp performance drops. This suggests LGCMT is relatively insensitive to the exact local window size, although optimal scene-specific selection, as used in our main experiments, can provide marginal gains, further validating our adaptive configuration strategy.

#### 4.3.6 Visualization.

We complement our quantitative evaluation with qualitative visualizations in real-world scenarios selected from the ETH and UCY test sets.

**Fig 6:**
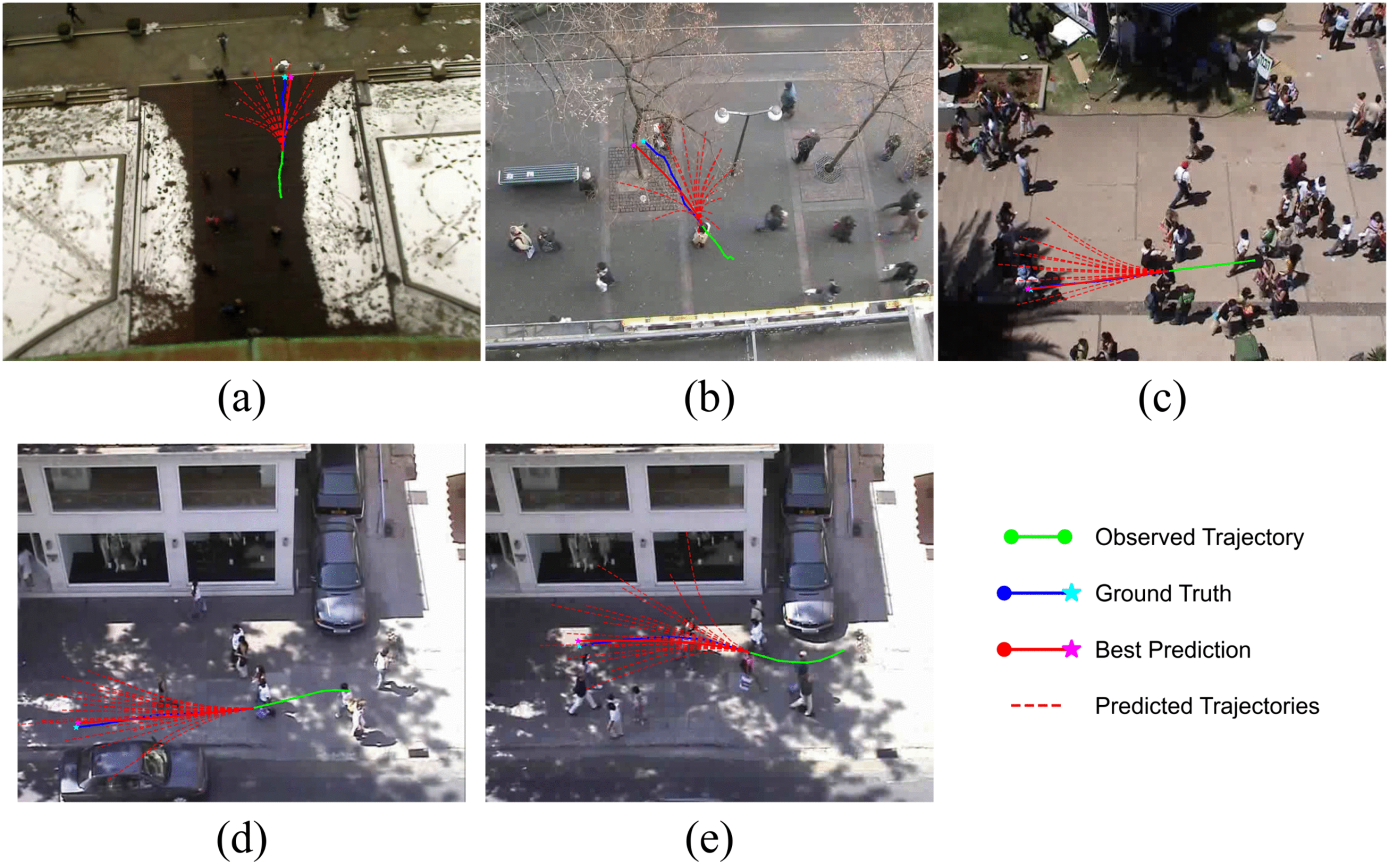
Multi-modal trajectory predictions on ETH/UCY scenes. **(A)** ETH, **(B)** HOTEL, **(C)** UNIV, **(D)** ZARA1, and **(E)** ZARA2. Observed trajectories are shown in green, ground-truth futures in blue, predicted trajectories are shown as red dashed lines, and the best prediction is highlighted in solid red.

Fig 6 showcases the diversity of predictions generated by LGCMT (*K* = 20). The visualization confirms that our library-guided approach can hypothesize varied yet plausible outcomes, effectively covering the ground truth. This ability to model distribution spread is crucial for capturing motion uncertainty in dynamic environments.

**Fig 7:**
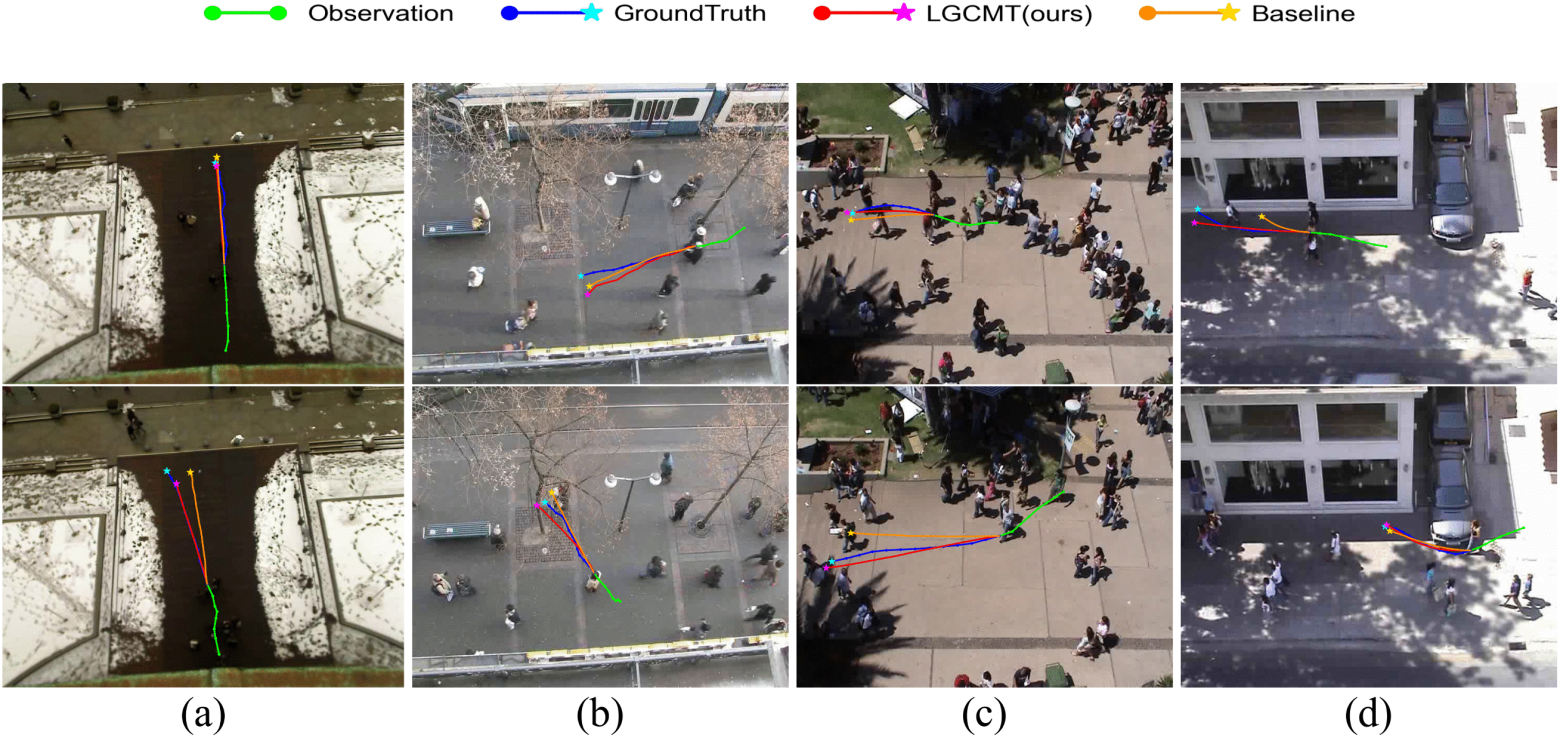
Qualitative comparison with a baseline method. **(A)** ETH, **(B)** HOTEL, **(C)** UNIV, and **(D)** ZARA. Observations are shown in green and ground truth in blue. LGCMT is shown in red and the baseline is shown in orange.

Furthermore, Fig 7 compares the best-predicted trajectory of LGCMT against the TUTR baseline [24]. In scenarios requiring complex maneuvers, such as navigating through crowds or approaching destinations, LGCMT demonstrates stronger adherence to the ground truth. Unlike the baseline, which may struggle with sudden directional changes, our model effectively captures fine-grained dynamics. Collectively, these visualizations validate that the proposed Local-Global Collaborative Encoder and NAR decoder successfully capture intricate pedestrian dynamics.

## 5 Discussion

The experimental results presented offer critical insights into the mechanisms underpinning LGCMT’s performance. The model’s effectiveness is rooted in the synergistic collaboration of its architectural components, which balance historical interpretation, structured guidance, and operational efficiency.

The ablation studies confirm that accurate prediction requires disentangling multi-scale temporal dynamics. The significant performance degradation observed when removing either the Global Context Encoder or the Causal Temporal Encoder underscores that neither short-term kinematics nor long-term intent is sufficient in isolation. By integrating these through specialized attention mechanisms (SCT-MSA and Cosine Similarity), LGCMT effectively captures the duality of pedestrian motion—reacting to immediate surroundings while maintaining a consistent destination.

Furthermore, the motion mode library proves to be a cornerstone for ensuring predictive diversity. By constraining trajectory generation to a learned set of behavioral patterns, the model effectively mitigates mode collapse and unrealistic path generation. This structured guidance, coupled with explicit social interaction modeling, ensures that predictions are not only diverse but also socially compliant. The strategic choice of the Library-Guided NAR decoder is further validated by the efficiency analysis. By eliminating the sequential bottleneck of autoregressive models, LGCMT achieves a dramatic inference speedup (approximately 30×) while maintaining high accuracy, confirming its suitability for latency-critical real-world applications.

Despite its demonstrated effectiveness, LGCMT offers several clear directions for further improvement. First, the reliance on a pre-constructed motion library means generalization is tied to the diversity of the training data. Future work could explore online adaptation or dynamic mode discovery to strengthen robustness under out-of-distribution behaviors.

Second, in terms of environmental context, the current framework relies solely on trajectory data and therefore does not explicitly model map semantics or obstacle geometry. Although the model can indirectly infer walkable regions from agents’ historical behaviors, it lacks explicit physical grounding to ensure collision-free predictions when facing complex static structures in highly organized scenes. This choice was made to prioritize computational efficiency and emphasize dynamic social interactions. Nonetheless, incorporating a lightweight, scene-centric branch to process semantic maps or occupancy grids would be a natural next step. Such an extension could improve generalization in navigation-intensive environments while largely preserving the inference efficiency of our architecture.

Finally, expanding the evaluation to broader domains is a promising direction. Beyond pedestrian dynamics, complex multi-agent interactions are prevalent in sports analytics, such as the NBA dataset. While distinct in input modality and team strategies, such scenarios share the need for modeling coupled spatiotemporal behaviors. Adapting LGCMT to handle these domain-specific constraints would be a valuable step toward testing the cross-domain generalizability of our local-global and library-guided approach.

## 6 Conclusion

This paper presented LGCMT, an efficient pedestrian trajectory prediction framework that adeptly captures the complex, multi-modal nature of human movement. The core strength of LGCMT lies in its innovative local-global collaborative encoder, which synergistically employs sparse causal temporal attention for local dynamics and cosine similarity attention for global patterns to construct a comprehensive historical representation. To address predictive diversity, we introduced a structured hypothesis mechanism guided by a motion mode library, ensuring the generation of varied yet plausible futures. By integrating social interaction modeling with an efficient non-autoregressive parallel decoder, LGCMT not only achieves competitive accuracy on the standard ETH/UCY benchmarks but also demonstrates robust scalability on the challenging SDD. These results confirm that LGCMT offers a compelling balance of performance and efficiency, making it highly suitable for practical deployment in real-world applications.
